# “That Little Bit of Time”: Transition-to-Hospice Perspectives From Hospice Staff and Bereaved Family

**DOI:** 10.1093/geroni/igab057

**Published:** 2022-01-18

**Authors:** Sarah H Cross, Janel R Ramkalawan, Jackie F Ring, Nathan A Boucher

**Affiliations:** 1 Sanford School of Public Policy, Duke University, Durham, North Carolina, USA; 2 Division of Palliative Medicine, Department of Family and Preventive Medicine, Emory University, Atlanta, Georgia,USA; 3 School of Medicine, Duke University, Durham, North Carolina, USA; 4 Transitions LifeCare, Raleigh, North Carolina, USA; 5 Center of Innovation to Accelerate Discovery and Practice Transformation (ADAPT), Durham VA Health System HSR&D, Durham, North Carolina, USA

**Keywords:** Bereavement, Caregiving—Informal, Death and dying, Qualitative analysis: Thematic analysis

## Abstract

**Background and Objectives:**

Many patients lack understanding of hospice services and their preparation for the transition to hospice at home may be insufficient. This study explored how hospice admissions staff and caregivers of hospice patients perceive the hospice admission process and the transition to hospice at home.

**Research Design and Methods:**

We conducted in-depth, semistructured interviews with 2 subgroups: hospice admissions staff (*n* = 15) and bereaved caregivers of former hospice patients (*n* = 20). We performed a 3-coder descriptive content analysis.

**Results:**

There were 4 overall themes: (a) issues relating to the referring/prehospice provider, (b) issues relating to hospital discharge/care transition home, (c) issues relating to the first touch of hospice, and (d) the impact of coronavirus disease 2019 (COVID-19) on hospice admissions. Patients are often referred to hospice without clear prognostic understanding, at times placing staff in the uncomfortable position of breaking difficult news. Stigma may make patients and families fearful of enrolling in hospice, and misconceptions about hospice are common. Caregivers emphasize the need for increased attention to their emotional needs. Staff revealed the emotional challenges they experience conducting admissions. Both staff and caregivers indicate that the transition to hospice is often emotionally and logistically burdensome, especially when discharging home from the hospital. Both subgroups report insufficient caregiver preparation for taking care of a dying patient at home, particularly regarding medication management. COVID-19 created challenges yet prompted innovative changes to hospice admission processes.

**Discussion and Implications:**

Findings demonstrate a need to improve the hospice admissions process, better supporting terminally ill patients and their families.

**Translational Significance:** The hospice admission process is an important transition point at the end of life. This study adds a unique perspective of the hospice admission—often understudied—and from the perspective of two critical constituencies, hospice admission staff and bereaved caregivers. Optimizing the hospice admission process from both the staff side and the health care consumer side may improve care transitions for patients discharging to home hospice from the hospital.

## Background and Objectives

Due to health care system fragmentation and frequent discontinuity of care, care transitions may result in poor clinical outcomes and are often perceived by patients and family as stressful and unsatisfactory (Coleman et al., 2003; Li et al., 2014; Mitchell et al., 2018). Care transitions in the final months of life remain common, even among patients who use hospice, a specialized type of palliative care for individuals with limited life expectancy (Teno et al., 2018; Wang et al., 2016). Individuals nearing the end of life may be especially likely to experience disrupted care given their substantial medical and social needs coupled with conditions due to aging. As more than 80% of hospice patients are aged 65 or older, improving the transition to hospice may be particularly impactful for the older adult population (National Hospice and Palliative Care Organization, 2020).

Patients are often referred to hospice following one or more hospitalizations (Waldrop & Meeker, 2012). Hospice referrals commonly occur from hospitals, and the likelihood of patients being referred to hospice by hospitalists has increased in recent years (Ankuda et al., 2017; Russell et al., 2017). Patients referred to hospice from hospitals may be more likely to use hospice for fewer than 7 days than patients referred from other sites (Furuno et al., 2020). Patients enrolling in hospice within the final week of life tend to be more functionally impaired and symptomatic than patients who elect hospice earlier in their disease course (Diamond et al., 2016). In 2018, nearly 28% of Medicare hospice beneficiaries used hospice for 7 days or less (National Hospice and Palliative Care Organization, 2020).

A rushed transition to hospice when patients are more debilitated and emotions are running high may carry a greater risk of miscommunication, medical error, and lack of attention to psychosocial needs of patients and caregivers alike (Moore et al., 2003; Murtaugh & Litke, 2002). Family members’ perception of a late hospice referral has been associated with poorer quality of care (Teno et al., 2007). As more Americans die at home and use hospice services, the need to improve the transition to hospice, particularly in the home setting, will grow (Cross & Warraich, 2019; National Hospice and Palliative Care Organization, 2020).

Gaps in the transition from hospital to hospice have been previously illuminated (Izumi et al., 2020). Research has examined hospice staff perceptions of patient and family desire for control when referred to hospice (Noh, 2019). Caregivers are an important determinant of the end-of-life experience, and their presence has been associated with an increased likelihood of dying at home (Ailshire et al., 2021). Yet, how caregivers perceive the hospice admission has not previously been explored. Although hospice eligibility criteria are set by Medicare, there is no widely standardized practice for hospice admissions (Center for Medicare and Medicaid Services, 2020). Some hospices have dedicated admissions staff, while others use a registered nurse (RN) case manager model where the same RN completes the admission and manages the patient’s care for the duration of their hospice stay (National Hospice and Palliative Care Organization, 2018).

How hospice services are presented to patients and caregivers during hospice admission visits may influence whether a patient chooses to enroll in hospice as well as their satisfaction with provided care (Vig et al., 2010). Optimizing hospice admissions offers an opportunity to better prepare patients and caregivers for hospice and potentially reduce negative outcomes during this important transition. This qualitative study sought to answer how do hospice staff and bereaved caregivers perceive the hospice admissions process.

## Research Design and Methods

### Design

This was an exploratory design comprised of semistructured interviews followed by a three-coder thematic analysis. Duke University Institutional Review Board (IRB) approved this study (#2020-0449).

### Participants

Hospice staff and bereaved caregivers were recruited from a hospice organization in Central North Carolina. The partnering hospice had an annual daily census of 641 in 2020 and a primary service area of eight counties. Hospice staff were eligible if they conducted hospice admissions, were older than the age of 18, could consent to research participation, could participate in an English language interview, and were willing to be audio-recorded. Caregivers were eligible if they had been the primary caregiver for an individual who used hospice services through the partnering hospice, were older than the age of 18, could consent to research participation, could participate in an English language interview, and were willing to be audio-recorded.

We employed a purposive sampling strategy. We asked our partner hospice to provide contact information for all hospice staff who conduct hospice admissions (nurses and social workers). S. H. Cross and J. R. Ramkalawan contacted the entirety of the hospice admission staff (*n* = 20) via email to provide information about the study and to schedule interviews if the staff member was interested in participation. To recruit caregivers, study flyers were included in the hospice bereavement service’s mailing sent to 2,700 caregivers of hospice patients who had died within the past 4–12 months. Flyers provided a brief study description, eligibility information, and researcher contact information. Potential participants were asked to call or email one investigator (S. H. Cross) if interested in participating. The interviewers (S. H. Cross and J. R. Ramkalawan) then contacted the interested caregiver to give further information on the study, confirm voluntary participation with verbal consent, and schedule the phone interview. Participants were provided a US$25 gift card as an honorarium for their time.

### Data Collection

Members of the hospice organization leadership team reviewed and refined the language of the semistructured interview guide questions designed by the research team. The final interview guide for hospice staff included questions about their experience of conducting hospice admissions, patient referral sources, the emotions that arise during admissions for both staff and patients/caregivers, and admissions that were “successful” as well as those that “did not go well.” Although this study was planned prior to coronavirus disease 2019 (COVID-19), as the pandemic was mentioned by staff during the first interview, we added an interview guide question asking how COVID-19 had affected hospice admissions practices for all subsequent staff interviews. Additionally, team reflection on the cadence, quality, and style of the first three interviews helped the team refine the guide and its application.

The interview guide for caregivers included questions about their referral to hospice, concerns they might have had about choosing hospice, the emotions experienced during the admission, and changes they would recommend be made to the admission process. Interviews were conducted by two researchers (S. H. Cross and J. R. Ramkalawan) between May and August 2020. Participants completed a brief demographic survey followed by a semistructured interview. All interviews were conducted by phone and audio-recorded. Interviewers documented summary observations in detailed memos following each interview. Average length of an interview with staff was 55 min and average length of an interview with caregivers was 41 min.

### Data Analysis

Each audio-recorded interview was transcribed using IRB-approved Temi.com and quality-checked by the interviewer. Our research team employed thematic analysis to identify, analyze, and report patterns found within the data (Guest et al., 2011). Our analysis began with the first interview in an effort to refine question framing, order, and use of probes to gather additional information or clarify responses. We used NVivo 12 (QSR International Pty Ltd. Version 12, 2018) for primary coding, data management, and illustrative quote abstraction following initial code generation using shared digital documents and iterative team meetings.

Two researchers (S. H. Cross and J. R. Ramkalawan) completed individual coding with intermittent discussion on three sets of three transcripts followed by confirmation coding by N. A. Boucher and then team discussions (Boeije, 2002). Once the codebook was generated, NVivo was employed by S. H. Cross and J. R. Ramkalawan to code three transcripts each with consultation from N. A. Boucher to determine accuracy. The team adjusted the codebook, and the changes were minor. Conflicts about data interpretation were resolved by consensus during team meetings. When the team had confidence in the application of codes, S. H. Cross and J. R. Ramkalawan completed coding on the remaining transcripts in NVivo. All authors discussed the emerging categories and themes at biweekly meetings. The lead researcher (N. A. Boucher) was continuously involved and leading the team in using a constant comparative approach—comparison of subsequent findings to previous findings to determine whether they were new categories, part of an existing category, or indicated a higher-level category—to strengthen the dependability of findings (Boeije, 2002). The postinterview memos, while not coded separately, were reviewed by the lead researcher in early analysis prior to transcription processing to help generate initial codes. The team also reviewed memos to gather real-time observations captured by interviewers. This contributed to the team’s data interpretation and highlighted areas to discuss in the analysis.

When the team determined that thematic saturation had been reached—when further observations and analysis were no longer yielding novel or discrete themes—we discontinued interviews (Guest et al., 2006; Saunders et al., 2018). Additionally, saturation was checked against the concept of “information power” earlier in the sample framing and recruitment process. Information power was determined prior to data collection by designing a projected adequate sample size, across two subsamples, for the relatively discrete study aim of examining the hospice admissions process. In the course of data collection, information power was determined through sufficiently detailed dialogue with participants achieved through rich and emotional interviews. Finally, thematic analysis directly focused on the admissions process and adjacent tensions (i.e., transitions of care, role of staff; Malterud et al., 2016; Saunders et al., 2018).

The research team’s combined expertise and experience in hospice social work, hospice, and hospital quality improvement administration as well as prior palliative care research allowed us to use *reflexivity* to probe our own understanding of the subject matter in team meetings (Eakin & Gladstone, 2020). For example, our team was able to articulate our understanding of hospice care processes, which create tensions for staff and caregivers alike, while acknowledging tensions may differ between hospices and in different hospice markets. This approach made us aware of our own professional *standpoints* that both helped and hindered understanding of the data (Eakin, 2010). For example, our collective knowledge of hospice processes from the staff’s point of view ran the risk of blinding us to the caregiver’s point of view. Through iterative team meetings, our team developed a shared understanding of the challenges faced by both respondent types. Furthermore, reflection by hospice admissions staff and senior leadership during a formal virtual session on their understanding of the anonymized, aggregated data improved our understanding of what we were hearing in interviews. Staff were able to clarify administrative challenges hampering optimal admissions processes, for example.

## Results

There were 35 participants: 15 hospice staff (Table 1) and 20 caregivers (Table 2). All hospice staff self-identified as female and most self-identified as White (87%). The mean staff age was 50 years. All but three of the staff members were RNs and 67% had a bachelor’s degree or higher. The mean length of experience in their clinical discipline was 17 years and the mean length of experience in hospice was 7 years. Seventy-nine percent of caregivers self-identified as female and 89% self-identified as White. The mean caregiver age was 59 years and 55% were the child of the hospice patient; the mean patient age was 78 years. Forty-two percent had obtained a graduate degree (masters or doctorate).

**Table 1.** Sociodemographic Characteristics of Hospice Admissions Staff (*N* = 15)

Variable	*N* (%)	*M* (*SD*); range
Gender		
Male	0 (0)	
Female	15 (100)	
Age (years)		50 (10.7); 32–64
30–40	4 (27)	
41–50	2 (13)	
51–60	6 (40)	
61+	3 (20)	
Race		
Black/African American	2 (13)	
White	12 (80)	
Latinx/Asian/Other	1 (7)	
Education level		
Associate degree	5 (33)	
Bachelor’s degree	7 (47)	
Master’s degree	3 (20)	
Clinical discipline		
Nurse	12 (80)	
Social worker	3 (20)	
Time as nurse/social worker (years)		17 (8.59); 7–31
<10	5 (33)	
10–20	6 (40)	
21–30	3 (20)	
31+	1 (7)	
Time in hospice (years)		7 (6.69); 1–27
0–3	4 (27)	
4–6	6 (40)	
7–10	2 (13)	
11+	3 (20)	
Time at partnering hospice (years)		5 (3.45); 1–13
0–3	5 (33)	
4–6	7 (47)	
7–10	1 (7)	
11+	2 (13)	

**Table 2.** Sociodemographic Characteristics of Bereaved Caregivers and Deceased Patients

Variable	Caregivers (*N* = 20)		Deceased hospice patients (*N* = 23)	
	*N* (%)	*M* (*SD*); range	*N* (%)	*M* (*SD*); range
Gender				
Male	5 (25)		10 (43)	
Female	15 (75)		13 (57)	
Age (years)		59 (12.7); 35–87		78 (14.5); 44–96
30–40	3 (15)		0 (0)	
41–50	0 (0)		1 (4)	
51–60	9 (45)		2 (9)	
61+	8 (40)		20 (87)	
Race				
Black/African American	1 (5)		1 (5)	
White	18 (90)		22 (95)	
Latinx/Asian/Other	1 (5)		0 (0)	
Education level				
High school graduate, GED	0 (0)		2 (9)	
Some college credit	2 (10)		7 (30)	
Trade/technical/vocational	0 (0)		2 (9)	
Associate degree	0 (0)		1 (4)	
Bachelor’s degree	10 (50)		7 (30)	
Master’s degree	5 (25)		4 (17)	
Doctorate	3 (15)		0 (0)	
Relationship to patient				
Spouse	9 (45)			
Child	11 (55)			
Time since death (months)				10.5 (13.7); 4–72
0–5			6 (26)	
6–8			9 (39)	
9–11			3 (13)	
12+			5 (22)	
Length of stay in hospice (days)				53.4 (83.7); 1–365
0–5			3 (13)	
6–15			10 (43)	
16–30			2 (9)	
31–90			4 (17)	
91+			4 (17)	

*Notes:* GED = General Educational Development. Three caregivers cared for multiple patients.

We identified four main themes encountered in the data: (a) issues relating to the referring provider or prehospice provider, (b) issues relating to hospital discharge and improving the transition to home, (c) issues relating to the first touch of hospice, and (d) the impact of the COVID-19 pandemic on hospice admission processes. Exemplar quotes for each subtheme are given in Table 3.

**Table 3.** Exemplar Quotes of Staff and Caregivers Perceptions of Hospice Admissions

Subtheme	Quotes
Theme 1: Issues relating to the referring or prehospice clinician	
Referral source and poor communication	We have families who’ve never heard the word hospice before … I’ve literally had a handful of patients who say, “well, the doctor never told me that” … families are blindsided.—Staff
	Eight days before he passed, he had a really bad night and [the nurse] said “You know, what’s going on with him is because it’s metastatic, it’s in his brain” … I had no idea … And I wish that I had known that because it would have helped me care for him.—Wife of a 75-year-old patient
Stigma and hospice misconceptions	Our impression with hospice had been, Oh, you’re put under hospice care because you’re dying and there’s nothing else to be done. It’s give up time … nothing could be further from the truth.—Wife of a 68-year-old patient
	A lot of times in hospitals they just get dropped the H-bomb and panic sets in for everybody.—Staff
Theme 2: Issues relating to hospital discharge and improving the transition to home hospice	
Palliative care improves process	There are [some] facilities that have actually a palliative care team there …. Those teams … come in and take the time with them to answer certain questions … it makes it so much better.—Staff
	If they’re coming from palliative care they usually have a lot more knowledge of the patient’s physical status … because they’ve kind of already been through those conversations.—Staff
Timing of the discharge	I thought once we said we want to go home on hospice, they would get the transport and we’d come home. And each doctor had to come in … the charge nurse … and then the hospice representative came and it was just so many people and so much paperwork … it’s not a good experience.—Wife of a 77-year-old patient
	Sometimes you have [discharges] where … [the family is] still at the hospital waiting for transport … the patient that was perfectly alert and oriented yesterday is now … having a pain crisis … you’re basically giving them a … “can you please sign the paperwork so that I can start getting somebody to run get some medicine” …. You have to keep your wits about you.—Staff
Pain management during the transition	We didn’t have enough pain medication from the hospital when we came home … [hospice was] going to come back in the morning …. I wish that before we had been left alone overnight that we had been more prepared.—Wife of a 77-year-old patient
	We have patients in the hospital on pain pumps and their final request is … to go home …. We used to have a process where … the nurse [would] hook up the pump for them to go home with … [now] if they discontinue the pump at the hospital, that patient could [have] an hour and a half where they haven’t had any pain meds.—Staff
Theme 3: Issues relating to the first touch of hospice	
Timing of the admission visit	There was a wasted day between my talking to the intake person … and the intake team coming up to interview me … the fact that she died four days later, I missed a day of having [hospice].—Husband of a 71-year-old patient
	I do think that sometimes they are too overwhelmed especially if they’re coming home from the hospital or just learned their diagnosis … the patient and family haven’t even had time to process.—Staff
Social worker presence	Having it be a dual visit with the social worker and the nurse … would have made it feel less sterile. Moving to hospice was such a defining decision … dealing with the emotional part first would have made dealing with the medical part much easier.—Wife of a 68-year-old patient
	I would love it if we could have a social worker present at every single admission visit …. I do feel like there are some visits where I don’t have one and I’m sure I’m doing this family a disservice.—Staff
Caregiver psychosocial needs	I remember [the admissions staff] being very clinical and they made it clear that they were just doing the intake … it felt very clinical. It didn’t feel personal.—Daughter of a 96-year-old patient
	I was trying to keep it together. Barely. I remember grabbing my mom’s hand a lot … it’s leading to someone very important in your life no longer being there and that is very challenging.—Son of a 67-year-old patient
Staff psychosocial needs	There are admissions that are particularly triggering … especially people with a cancer diagnosis that have small children …. My mother had cancer three times so I’m always sensitive to that situation.—Staff
	I remember it being really quite hard when I first started, I felt like I was tearing up and … trying not to cry in the actual admissions …. I had to internally deal with that for quite some time.—Staff
Setting expectations	Everything you do … sets them up for failure or to have a positive experience with us … I’m the person that they’re establishing trust with and if I am not here for them in that little bit of time, then they’re just gonna start on the wrong foot with our organization.—Staff
	You sign up with hospice and think, “Oh boy, here comes some help.” And then it didn’t really come except with the certified nursing assistants would come and be here for 45 minutes, then they’re gone … it was kind of a letdown that they weren’t more involved.—Husband of a 56-year-old patient
Medication management at home	It’s a big deal to have a box of morphine and lorazepam in your refrigerator … one’s very much aware that these are narcotics and that they can be dangerous.—Daughter of a 92-year-old patient
	If no one has given morphine ever, they’re going to be hesitant most likely and they’re going to need further education about opioids and pain management.—Staff
Theme 4: The impact of the coronavirus disease 2019 (COVID-19) pandemic on hospice admission processes	
Improvements as a result of COVID-19	We have a support nurse role that just came into play during coronavirus, and that has been so helpful … she’s helping all the admissions nurses who are in the field that day.—Staff
	Especially now in COVID, a lot of people are finding the best thing about hospice. They have somebody go in and look at their loved one and make sure they’re all right. And that’s huge because they’re …. They’re shut out of the facility.—Staff
Challenges as a result of COVID-19	If it’s a dementia patient or a hard of hearing patient, it’s very difficult for them to hear you or to read your mouth or to figure out what you’re saying cause they’re looking for nonverbal cues and just how they cope with their communication issues.—Staff
	It’s awful … you’ve literally just sat here and listened to them pour their hearts out and given them confidence that they can do this and that everything’s going to be okay … at the end you can’t even shake their hand.—Staff

### Issues Relating to the Referring or Prehospice Provider

#### Referral source and poor communication

According to hospice staff and caregivers in our sample, patients are often not properly prepared for hospice by health care providers ahead of a referral to hospice. Many caregivers indicated that they and the patient lacked knowledge of what to expect with their disease course and in some cases were not aware that the prognosis was terminal. As a result, hospice staff at times found themselves having to break difficult news to patients during the admission.

Several hospice staff noted that patients referred to hospice by their oncologist tend to have greater awareness of their disease trajectory and a stronger understanding of hospice. Both hospice staff and caregivers identified communication challenges with patients’ primary clinicians, a likely contributor to the limited prognostic awareness referenced.

#### Stigma and hospice misconceptions

Many caregivers described their reluctance to use hospice because of stigma and fear that hospice would hasten death. Staff also echoed these concerns. The confidence of several caregivers was not restored by the prehospice care teams. They noted language used to describe the transition to hospice was of “giving up.” Some caregivers also noted that they were referred to hospice with little understanding of why or without an explanation of hospice services. One caregiver recalled desiring “more insight and reassurance” from the doctor that “we’re not telling you you’re going to die tomorrow” when hospice was suggested. Staff shared that, in their experience, individuals with whom they meet often have inaccurate preconceived ideas of what hospice is, noting that some referring providers do not adequately explain hospice, leaving patients and families with their misconceptions; however, one caregiver noted that despite hospice discussions with her spouse’s doctor, they were not receptive to the idea because of their “negative concept” of hospice.

One staff likened the mention of hospice to an “H-bomb” dropped on patients and families that resulted in panic with little prior preparation or explanation before the hospice admissions staff were able to see the patient and detail the benefits of hospice.

### Issues Relating to Hospital Discharge and Improving the Transition to Home Hospice

#### Palliative care improves process

Hospice staff noted that the hospital discharge process can often be emotionally and logistically burdensome for patients and caregivers. Staff reported that the transition from hospital to home hospice tends to be easier when patients have been involved with inpatient palliative care and noted the disparate availability of these teams among the hospitals from which they receive referrals, particularly rural ones.

#### Timing of the discharge

Several staff and caregivers shared their frustrations with the slow process of hospital discharges once hospice has been decided upon. Staff noted the multiple moving parts that must be managed to ensure all is in place for a new patient, particularly when the patient’s medical condition is more fragile or has changed. Some caregivers reported that they were discharged home before all durable medical equipment and medications had been arranged for delivery by hospice. Furthermore, we interviewed several staff and caregivers who believed the patient and family had not been able to sufficiently settle in at home or process their decision to use hospice before staff came to admit the patient to hospice.

#### Pain management during the transition

Participants also revealed gaps in pain management during the transition from the hospital to home hospice. One caregiver, for example, indicated that she lacked sufficient pain medication to treat her husband between the hospital discharge and the hospice admission which had been scheduled for the following day. Staff also echoed concerns about patients’ pain management during this critical time. One staff member noted that they were no longer able to set up pain pumps for patients ahead of hospital discharge, which particularly disadvantaged patients who resided far away from the hospital.

### Issues Relating to the First Touch of Hospice

#### Caregiver psychosocial needs

Interviewees broadly noted a hospice admission may be fraught with emotion for the patient and family. Caregivers spoke about the emotions that arose for them during the admission. For example, one caregiver recalled that having to complete paperwork took her away from the side of her dying spouse, causing her great distress.

#### Staff psychosocial needs

Staff also discussed the emotional challenges of conducting hospice admissions. Some indicated they adopt a more business-like manner in order to remain focused and emotionally detached, while others shared that they had struggled to manage their feelings when beginning hospice work. Staff whose loved ones had experienced a particular illness reported being triggered at times by patients who had that same illness; others noted that working with younger patients could be especially distressing.

#### Social worker presence

Both staff and caregivers emphasized the value of social worker presence during the admission. One caregiver felt as though hospice staff acted more clinical than personal and noted that having had a social worker at the admission would have better enabled her to deal with the medical aspects of the admission. Several admissions nurses indicated their lack of training in psychosocial issues and felt better able to support patients and caregivers during an admission when partnering with a social worker.

#### Timing of the admission visit

Some staff members voiced concern that their visiting a patient soon after hospital discharge or learning about a terminal diagnosis may add to the patient and family’s emotional strain. Staff discussed trying to balance the imperative to thoroughly educate the patient and/or caregiver with the need to allow the patient to rest after a hospital stay or perhaps to process upsetting information. Some caregivers felt that hospice admissions staff came out too soon after returning home from the hospital. However, a husband caregiver of a patient who died less than a week after being admitted to hospice regretted that the admission did not occur sooner after the hospice referral was made.

#### Setting expectations

Our interviews also indicated the importance of ensuring that patients and caregivers have an accurate understanding of what hospice services do and do not entail. A number of caregivers shared that although they wanted to care for their loved one at home, they were not initially aware of or prepared for how challenging providing care in the home would prove to be. Compounding these challenges was the fact that many caregivers did not realize that the family is responsible for the majority of patient care even when hospice services are in place—some felt let down when they realized this.

#### Medication management at home

Several caregivers noted fear and uncertainty about managing the patient’s medications at home. One caregiver, who is a nurse, discussed her hesitancy about giving prescribed morphine to the patient and noted her fear of that drug persisted despite her nursing background. Staff confirmed that such concerns are common and that caregivers often need repeated education about medication administration and safety as well as combating morphine stigma.

### Issues Relating to the Impact of the COVID-19 Pandemic on Hospice Admission Processes

#### Improvements as a result of COVID-19

While COVID-19 posed significant challenges for staff contact with patients and their families, some staff reported the pandemic had forced improvements in processes they hoped would last. One staff noted that efforts to reduce the number of personnel in the field had prompted the creation of new roles and streamlined procedures, such as a support nurse who helps field nurses with ordering medication and equipment. Additionally, hospice staff became a more important point of contact for patients as they were among the few able to visit patients regularly in facilities and at home.

#### Challenges as a result of COVID-19

More commonly, participants noted the ways in which COVID-19 had disrupted usual care. Most staff lamented the absence of personal connection forced by the pandemic and noted how masks and face shields hindered conversations, reading facial expressions, and hugs. To limit the amount of time spent in the home, staff provided more information over the phone or via video; as a result, admissions visits were often shorter. Although staff believed that most patients adapted well to virtual care, they noted that some patients were less comfortable with the new technology. Furthermore, the lack of in-person visits, in addition to the use of face masks, presented communication barriers to some patients who had dementia or hearing impairments. Emotionally supporting patients and families was also difficult to achieve with the imposed distancing and wearing of personal protective equipment. As the patients our caregiver participants cared for died prior to COVID-19, caregivers could not speak to the impact of the pandemic on hospice admissions; however, some regretted that the pandemic had suspended in-person bereavement support groups normally provided postdeath to caregivers.

## Discussion and Implications

The hospice admission is the front door to hospice services. Patients’ and families’ perceptions of the admission process set the stage for the hospice stay and shape their care use. Hospice admissions staff, often the first hospice representative that patients and families encounter, may likewise influence the hospice experience. Our semistructured interviews reveal how this crucial encounter is viewed by caregivers and hospice staff and highlight areas for improving the transition to hospice.

In line with prior research, both hospice admissions staff and caregivers indicate that patients are often referred to hospice with the explanation that hospice is “extra help” but without a clear understanding of their prognosis or what hospice services entail (Cagle et al., 2016). Some clinicians are uncomfortable with end-of-life discussions, and though not all patients want to know their prognosis, this knowledge may help caregivers arrange appropriate care and support (Glare & Sinclair, 2008). While many palliative care clinicians receive specialized education in communication and delivering difficult news as part of fellowships, hospice staff may not receive this type of training as one assumes that these conversations had occurred prior to hospice referral. Patients who better recognize their limited life expectancy may be more willing to shift from curative treatment to comfort-focused care (Vig et al., 2010; Weeks et al., 1998).

In our sample, many caregivers found communication with the patient’s primary clinicians ahead of a referral to hospice to be poor. Many hospice staff indicated that patients referred to hospice by oncologists often have a more thorough understanding of both their disease trajectory and hospice services; however, some participants who were caregivers for patients with cancer reported having poor prognostic understanding ahead of the hospice referral. Although survival predictions are often more accurate in cancer and patients with terminal cancer may better recognize their limited life expectancy, it is unknown whether the quality of clinician–patient communication about hospice varies according to the patient’s disease (Waldrop & Meeker, 2012; Warraich et al., 2016). Regardless of diagnosis, previous research has indicated the need for more family and dying person involvement in the decision to use hospice as opposed to it being decided predominantly by clinicians (Casarett et al., 2004; McFarlane & Liu, 2020).

According to both staff and caregivers, stigma around death/dying makes many patients and families fearful of enrolling in hospice; misconceptions and lack of knowledge about hospice and palliative care more broadly are common (Boucher et al., 2018; Cagle et al., 2016). The misconception that hospice means imminent death may contribute to heightened emotions often experienced by patients and caregivers during hospice admissions. Patients’ primary providers play integral roles in patient and family education about hospice clarifying inaccurate beliefs; however, many health care professionals themselves lack accurate understanding about hospice (Snyder et al., 2013).

Once hospice has been selected, both staff and caregivers indicate that the transition to hospice can be both emotionally and logistically burdensome, particularly when discharging home from the hospital. Some of the staff whom we interviewed shared that the discharge from hospital to home was more seamless when patients had been involved with hospital palliative care and acknowledged the lack of palliative care in some hospitals. Research suggests that inpatient palliative care may improve hospital-to-home transitions for patients nearing the end of life and reduce negative outcomes such as hospital readmission (Saunders et al., 2019; Scott et al., 2020). However, disparities in access to inpatient palliative care exist. Rural hospitals are less likely to provide palliative care than urban hospitals and this is the case in North Carolina (Fink et al., 2013; Johnson et al., 2021).

Our interviews also highlighted the need for more thoughtful scheduling of admissions visits, especially when patients are transitioning home from the hospital. Medicare guidelines require that nurses complete their initial assessment within 48 h of hospice election and that the comprehensive assessment (completed by all members of the interdisciplinary team) be completed within 5 days of hospice election (Centers for Medicare & Medicaid Services, HHS, 2008). While some hospices may have nurses complete both the hospice election paperwork and the initial assessment, other agencies have nonclinical liaisons who assist patients and caregivers with the hospice election forms. Therefore, patients and caregivers typically must see multiple staff members in the days after choosing hospice services when they may still be grappling with a new terminal diagnosis, have just experienced a tiring hospital stay, or need to ensure the availability of other family members. Hospice staff should better assess patient/caregiver readiness for additional staff visits when enrolling a patient into hospice.

Many patients are, of course, referred to hospice when death is imminent, and getting admissions assessments and paperwork completed promptly is of utmost importance. We recognize that it may at times be difficult for hospice staff to balance their need to meet Medicare guidelines and to accommodate patient and caregiver preferences. As our interviews indicate, caregivers’ views on the timing of admissions visits were not uniform. Of note, previous research suggests that increased competition among hospice providers has likely influenced hospice admissions practices (Dolin et al., 2017). Some hospices may rush admissions out of concern that they could lose the referral to another hospice—pressure that may inadvertently be passed on to the patients and caregivers.

In our interviews, caregivers emphasized the need for increased attention to their emotional needs during the admission process and some bemoaned the focus on the *patient’s* clinical needs. Family members of hospice patients and caregivers may underestimate the emotional and spiritual support they need in advance of caring for a dying loved one (Casarett et al., 2004). Similarly, some admission nurses emphasized the specific skill set that social workers bring and wished that social workers could join them for all hospice admissions. Having social workers present for admissions may be infeasible given staffing limitations, as well as pressures to maximize productivity and minimize costs. Furthermore, there are known staffing shortages in palliative care, though less is known about the state of the hospice workforce (Kamal et al., 2017, 2019). Additionally, staff revealed the emotional challenges they themselves experience when conducting admissions. Research reveals substantial burnout among palliative clinicians and hospice staff likely experience it as well (Kavalieratos et al., 2017). Alleviating this burden is key to the sustainability of the hospice and palliative workforce.

Our interviews also showed that caregivers are often insufficiently prepared for taking care of a dying patient in the home setting. Some caregivers voiced regret that they did not know how difficult the role of caregiver would be and wished they had been better prepared during the admissions process. Patients/caregivers are often unaware that despite the round-the-clock availability of hospice, the family is responsible for the majority of hands-on care. Several caregivers reported feeling let down when they realized the limitations of the hospice benefit. Multiple staff members echoed these concerns, noting that families are often not prepared for the amount of caregiving involved in bringing a loved one home, particularly when the caregiver is older or has limited additional support. Additionally, both caregivers and hospice staff shared that patients and families are often uncomfortable managing medications at home. Previous research with caregivers of home hospice patients reveals that caregivers and families need improved education and support regarding opioid dosage and use (McFarlane & Liu, 2020). Misconceptions about pain medications in hospice are common and stigma around morphine may make caregivers apprehensive about giving medicine out of fear of opioid dependence or hastened death (Ho et al., 2020; Lau et al., 2009; Noh, 2019; Wegier et al., 2020).

Regarding the effects of COVID-19, we found that staff felt that personal protective equipment and the need to maintain distance affected the interpersonal experience making the hospice admission feel less personal. This is in line with recent literature identifying the multiple ways in which COVID-19 has negatively affected the provision of hospice and home palliative care (Franchini et al., 2021; Rogers et al., 2021). Despite these many challenges, staff reported that the pandemic had prompted improvements to some processes. A multinational survey of specialist palliative care providers indicated that COVID-19 has forced practice changes, including greater use of technology and streamlining of services, many of which may be retained long term (Dunleavy et al., 2021).

While this is a unique study looking at a critical pivot point at the end of peoples’ lives through the eyes of hospice staff and bereaved family members, there are limitations. Our participants are from one hospice agency; a broad perspective of the hospice admission experience is not possible to characterize here. Also, caregiver participants who contacted us in response to our recruitment outreach were mostly White and highly educated. Though this study does not include perspectives from a diverse population, it mirrors the characteristics of the general hospice population that is also majority White (National Hospice and Palliative Care Organization, 2020). Racial disparities in health research participation are well established; however, dedicated efforts to recruit and enroll research participants from racial and socioeconomic groups must be prioritized in order to reduce disparities in palliative care and hospice use (George et al., 2014; Johnson, 2013).

Improving the care transition experience is especially important for patients discharging to home hospice from the hospital. The hospice admission is a key point in this process, and our findings highlight several areas where the hospice admissions process could be improved to enhance this care transition. Hospices should assess patients’/caregivers’ understanding of prognosis from referral sources when a referral is made. Referring providers should have a more active role in educating and emotionally preparing patients ahead of a hospice referral. Despite greater awareness of hospice, there is a continued need to provide accurate education about hospice to the public. Greater use of social workers during hospice admissions visits may ensure greater emotional support for patients and caregivers alike. Efforts to streamline the hospital discharge process for patients who will be discharging to hospice may increase patient and family satisfaction and smooth continuity of care. Hospital staff and hospice admissions staff should be frank with patients and caregivers about the challenges of dying at home. Hospice procedures should include robust assessment of patient/caregiver expectations and comfort with medication use. Finally, COVID-19 has altered clinical care, challenging providers, bereaved caregivers, and patients as well as resulting in the streamlining of some care practices. Continued research should assess the impacts of the pandemic on care provision as well as the well-being of providers, caregivers, and patients.
